# Scaling properties of food flow networks

**DOI:** 10.1371/journal.pone.0199498

**Published:** 2018-07-10

**Authors:** Megan Konar, Xiaowen Lin, Benjamin Ruddell, Murugesu Sivapalan

**Affiliations:** 1 Department of Civil and Environmental Engineering, University of Illinois at Urbana-Champaign, Urbana IL, United States of America; 2 School of Informatics, Computing, and Cyber Systems, Northern Arizona University, Flagstaff AZ, United States of America; 3 Department of Geography and Geographic Information Science, University of Illinois at Urbana-Champaign, Urbana IL, United States of America; Uniiversity of Padova, ITALY

## Abstract

Food flows underpin the complex food supply chains that are prevalent in our increasingly globalized world. Recently, much effort has been devoted to evaluating the resources (e.g. water, carbon, nutrients) embodied in food trade. Now, research is needed to understand the scientific principles of the food commodity flows that underpin these virtual resource transfers. How do food flows vary with spatial scale? To address this question, we present an empirical analysis of food commodity flow networks across the full spectrum of spatial scales: global, national, and village. We discover properties of both scale invariance and scale dependence in food flow networks. The statistical distribution of node connectivity and mass flux are consistent across scales. Node connectivity follows a generalized exponential distribution, while node mass flux follows a Gamma distribution across scales. Similarly, the relationship between node connectivity and mass flux follows a power law across scales. However, the parameters of the distributions change with spatial scale. Mean node connectivity and mass flux increase with increasing scale. A core group of nodes exists at all scales, but node centrality increases as the spatial scale decreases, indicating that some households are more critical to village food exchanges than countries are to global trade. Remarkably, the structural network properties of food flows are consistent across spatial scales, indicating that a universal mechanism may underpin food exchange systems. In future research, this understanding can be used to develop theoretical models of food flow networks and to model food flows at resolutions for which empirical information is not available.

## 1 Introduction

We live in an increasingly global society, in which food commodity transfers enable production and consumption activities to be separated in space via complex supply chains [1, 2]. Here, we refer to the movement of food commodities from one location to another as ‘food flows’, reserving the term ‘food trade’ for the international exchange of food commodities between countries. Recently, much research has evaluated food trade [3–6], particularly the resources embodied in food trade, such as water, carbon, and nutrients [7–9]. However, we know relatively little about food flows at smaller spatial scales, such as within nations or cities. This is largely due to a lack of available data on food flows at smaller spatial scales. Research is needed to understand food flows across spatial scales in order to uncover the scientific principles behind food flows. To this end, we present an empirical analysis of food flows across the full spectrum of spatial scales: global, national, and village.

Despite the importance of food flows at local to global scales, we do not understand how they are similar or different depending on the scale of analysis. Evaluating similarities and differences in food flows across spatial scales may yield important insights into their underlying structure and function. Scaling analyses have yielded insight into the underlying observed patterns and processes in ecology [10], biology [11], hydrology [12], and urban metabolism [13], among others. Within many local communities, it is common practice for some households to share food and other goods with those households that have experienced a negative shock event, such as a death in the family, outbreak of pests, or drought event [14, 15]. These household resolution exchanges of food within a village represent the smallest spatial scale of social food exchanges. At the global scale, nations trade food commodities according to their comparative advantage, resource endowments, food production and trade policies, and international politics. Food flow networks are driven by human behavior across spatial scales, so the mechanisms leading to food flows relate to the food production, consumption, and trade decisions of people. In this way, the fundamental human behavior driving food flows (e.g. cooperation, kinship, risk sharing, economic welfare maximization, etc.) may leave its signature on the patterns of food flows across spatial scales.

Recent work has made significant strides in empirically describing [3, 16, 17] and modeling food flows [18]. Future food flow modeling would benefit from enhanced understanding of the empirical properties of food flows across spatial scales. In this paper, we empirically characterize food exchange networks across three dramatically different spatial scales: global, national, and village. For the village scale, we obtain data on household resolution donations of food and non-food commodities within three Alaskan villages [15]. Households exchange food items and other necessities with their neighbors and other households in their community in response to the heterogeneous distribution of availability and need, driven by a sense of kinship and reciprocity [15, 19]. For the national scale, we obtain data on commodity flows between Commodity Flow Survey zones of the United States [20]. The heterogeneous distribution of production and consumption at the national scale are additionally constrained by critical and inter-dependent infrastructure, such as the national transportation system [21, 22]. For the global scale, we obtain data on international commodity trade from the United Nations Commodity Trade Statistics Database (COMTRADE) [23]. Heterogeneity in production and consumption and infrastructure constraints are again important, along with the additional forces of trade policies and global commodity markets [24].

Network statistics provide a coherent framework for comparing complex systems across a range of scales [25]. Examining how food flow networks change with the scale of description is essential in order to elucidate mechanisms underlying observed patterns, as well as for simplification, aggregation, and scaling (i.e., the relationship of variables with some measure of size or scale). Additionally, understanding network characteristics enables us to gain insight into the potential susceptibility of these interconnected commodity flow systems to shocks [6]. The network structures of food flow systems will provide a signature of their vulnerability and resiliency to disturbance [3, 6], with important implications for embodied energy and water resources [26].

Network properties of food and embodied resource transfers have been investigated at the global [3, 27, 28], national [17, 29], and urban [30, 31] scales. However, the network properties of the commodity flows that underpin virtual resource transfers have not been compared consistently across spatial domains. Similarly, comparisons between food and non-food flows, flow directionality (i.e. origin, destination, undirected), and unit of measurement (i.e. mass, value) have not been consistently evaluated. Here, we quantify food commodity flow network properties across the full spectrum of spatial scales. Importantly, we compare food flows networks with non-food commodity flows, by flow direction, and by measurement unit. The primary question we address is: How do food flow networks vary with spatial scale? We also address the following questions: How are food and non-food flow networks different? How does flow direction impact network properties? How does the unit of measurement impact network properties? We present our methods in Section 2. Our results are presented in Section 3. We conclude in Section 4.

## 2 Methods

Here, we describe the methods used in this paper. First, we detail the data sources of food flows at each spatial scale. Second, we explain the network statistics and distributions that we used to quantify these food flows.

### 2.1 Commodity flow data across scales

**Fig 1:**
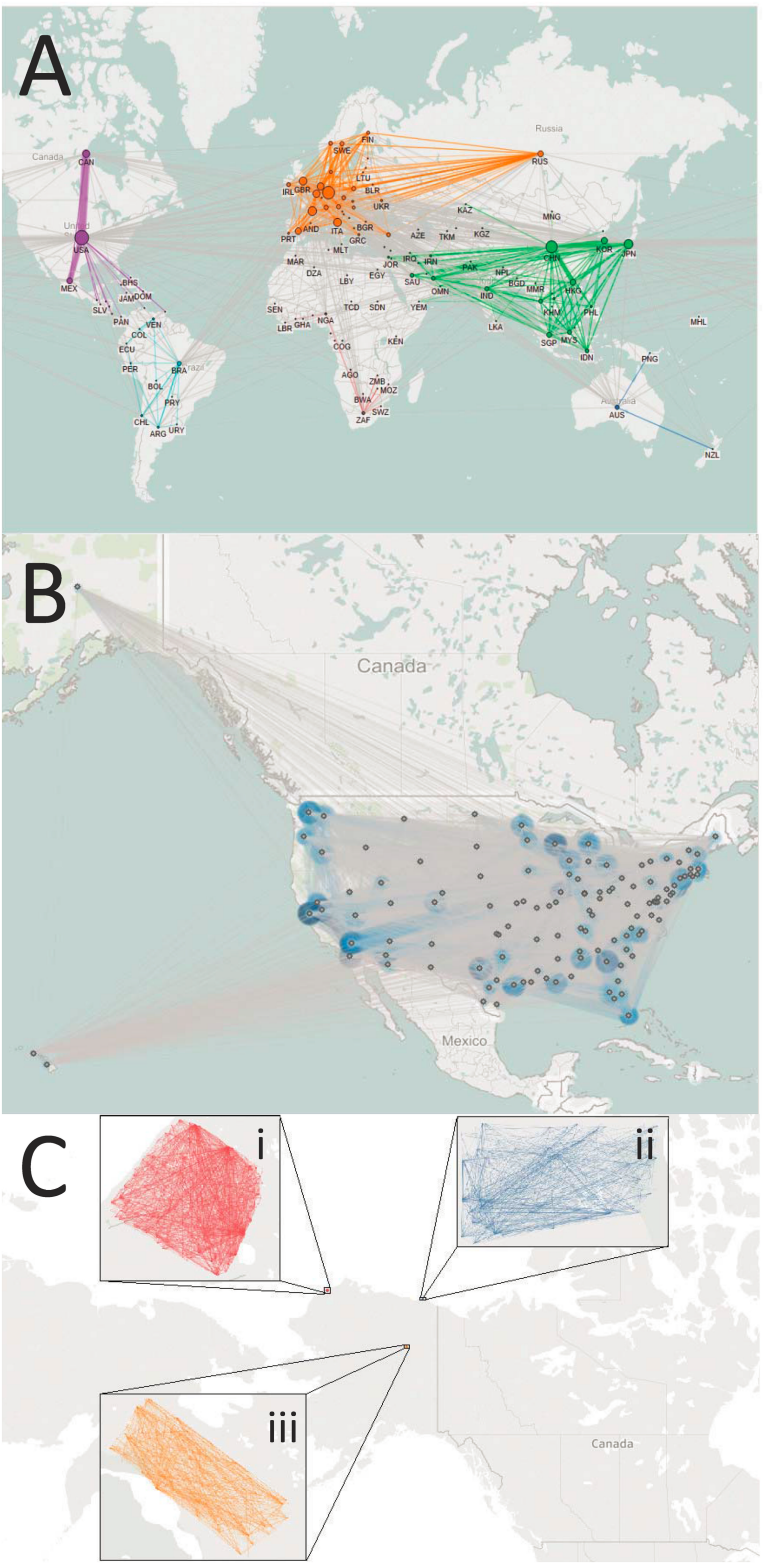
Maps of food flow networks for all spatial domains. (A) Map of international food trade between countries. (B) Map of food flows between Commodity Flow Survey areas in the United States. (C) Map of food exchanges between households in three Alaskan villages: (i) Wainwright, (ii) Kaktovic, and (iii) Venetie. Note that network illustration for villages is for visualization purposes only, as geographic locations of households are not provided. In (A) and (B) bubbles are scaled according to the total mass flux of food by node.

We obtain empirical information on commodity flows at three spatial scales: ‘global’, ‘national’, and ‘village’. ‘Global’ data refers to international commodity trade between 240 countries for the year 2009. International trade data comes from COMTRADE [23] and is mapped in Fig 1A. ‘National’ commodity flow data is for the United States and is obtained from the Commodity Flow Survey (CFS) for the year 2007 [20]. The CFS dataset breaks the United States into 132 CFS Areas. A map of commodity flows within the United States is provided in Fig 1B. ‘Village’ scale data on commodity flows are available for all households for three Alaskan villages: Wainwright, Kaktovic, and Venetie (locations shown in Fig 1C). Data on village scale commodity exchanges are available for the years 2009 and 2010 [15] and are mapped in Fig 1C.

Importantly, each dataset provides information on bilateral transfers between all nodes within the spatial domain, eliminating selection bias. Unfortunately, the data across these three scales are not uniform. First, the time period is different. Both village and global data are available for the year 2009. However, the national data comes from CFS, which is only collected every 5 years. We have selected 2007, which is the closest available year to 2009. Second, the commodity categories are different across spatial scales. To handle this issue, we lump commodities into two broad categories: ‘food’ and ‘non-food’ commodities. This broad categorization of commodities should alleviate some of the inconsistencies across datasets.

Categorizing commodity flow data as food and non-food commodities enables us to distinguish the unique aspects of food commodity flows across scales. At the global scale, Harmonized System (HS) codes 1 to 24 are food commodities, while non-food items are HS codes greater than 24. For the national scale, the Standard Classification of Transported Goods (SCTG) codes 2, 3, 4, 5, and 7 are food commodities and non-food commodities are all other SCTG codes. Village scale data on commodity donations are all food, with the exception of reports of equipment, cash, gas, ammunition, and donations of labor [15]. Global and national commodity flows are provided in both mass [kg] and value [$], while village flows are only available in units of mass. We weight commodity flows in these primary units, as they are most likely to be linked to the underlying heterogeneity and driving mechanisms.

The average area of a country in the global trade system is 5 x 10^11^ m^2^ [32]. The area of the United States is 9,147,420 km^2^ [32], and there are 132 CFS Areas. So, we estimate that the average area of a CFS Area in the United States is 7 x 10^10^ m^2^. The average size of an American house is 222 m^2^ [33], which we assume is similar to the size of households in the three Alaskan villages. So, node size in the three systems varies across roughly 10 orders of magnitude.

### 2.2 Network analysis of commodity exchanges

For each commodity flow network, the nodes are the units exchanging commodities and the links are the bilateral commodity flows. The full specification of the data allows us to characterize weighted and directed networks. From these networks, we can also evaluate their simpler unweighted and undirected counterparts. Here, we consider both unweighted/weighted and undirected/directed networks in order to consistently assess flow directionality and flow intensity across spatial scales.

The weighted, directed matrix (*W*_*D*_) contains elements (*w*_*i*, *j*_) that provide the weighted, link-level flows from node *i* to *j*. Each element *w*_*i*, *j*_ has a value >0 when a commodity flow exists from node *i* to node *j*. The unweighted version of *W*_*D*_ is referred to as an adjacency matrix (*A*_*D*_), which is a binary matrix in which only connectivity information is present. Each element (*a*_*i*, *j*_) is equal to 0 when no connection exists from node *i* to *j* and equal to 1 when there is a connection from node *i* to *j*. When direction is not taken into account, *A*_*D*_ is a symmetric matrix (*A*_*U*_) in which each element *a*_*i*, *j*_ is equal to 0 when no connection exists between node *i* and *j* and equal to 1 when there is a connection between node *i* and *j*. Note that information on the flow direction between node *i* and *j* is not available in *A*_*U*_ [34].

Network density refers to the number of links that exist in the network as a fraction of the total potential number of links. Density is a global network property and is measured by *p* = *M*/[*N*(*N* − 1)], where *M* is the number of links and *N*(*N* − 1) is the number of possible links [34]. First order network properties examine the attributes of individual nodes in the network. Node degree (*k*) is a fundamental network property that measures the connectivity of each node. For directed networks, there are two kinds of degree: out-degree ($k_{i}^{out}$) and in-degree ($k_{i}^{in}$). The number of outgoing links is defined as $k_{i}^{out}=\sum_{j}a_{i,j}$, while the number of incoming links is defined as $k_{i}^{in}=\sum_{j}a_{j,i}$. The total degree is defined as $k_{i}=k_{i}^{out}+k_{i}^{in}$ and measures the total number of commodity exchange partners of each node. For undirected networks $k_{i}^{out}$ and $k_{i}^{in}$ are equivalent. Node strength (*s*) takes weight into account by summing the weights assigned to each node’s links. For directed networks, $s_{i}^{out}=\sum_{j}w_{i,j}$ and $s_{i}^{in}=\sum_{j}w_{j,i}$. Total node strength measures the total value of commodity flows of each node, $s_{i}=s_{i}^{out}+s_{i}^{in}$. For undirected networks, $s_{i}^{out}$ is equivalent to $s_{i}^{in}$ [34].

Higher order network properties examine attributes of the neighborhood of nodes in the network. Node clustering (*c*) describes the propensity of nodes in the network to form closed triangles [35]. This is a classic measure of the ‘cliquishness’ of a social network. Node betweenness centrality (*B*) is calculated as $B=\sum_{i,j}\frac{\sigma (i,u,j)}{\sigma (i,j)}$, where *σ*(*i*, *u*, *j*) is the number of shortest paths between nodes *i* and *j* that pass through node *u*, *σ*(*i*, *j*) is the total number of shortest paths between *i* and *j*, summed over all pairs *i*, *j* of nodes [34]. *B* is normalized by 1/(*N* − 1)(*N* − 2) such that ∈ [0, 1] [36]. Directed paths are used to calculate directed *B* and undirected paths for undirected *B*. *B* measures the importance of a node to the overall network structure.

## 3 Results and discussion

Here, we provide the results of our empirical analysis of both food and non-food flows at global, national, and village spatial scales. First, we map and determine the global properties of flow networks. Second, we determine the statistical distributions that best fit the networks across spatial scales. Third, we characterize the parameters of the statistical distributions across all scales.

### 3.1 Summary statistics

Fig 1 provides a map of food flows for each spatial domain of analysis. Panel A maps international food trade between countries. Note that world regions are color coded so that regional food trade can be more clearly observed. Panel B maps sub-national food flows within the United States. Food flows between the 132 CFS areas are depicted. Bubbles in Panel A and B are scaled by the total mass flux of food for each node. Panel C illustrates food flows between households in three Alaskan villages: (i) Wainwright, (ii) Kaktovic, and (iii) Venetie. However, geographical information is not available for households at the village spatial scale. So, maps of village food flow networks are provided for illustration purposes only and are not geographically accurate.

Table 1 provides summary statistics for the three spatial domains. There are 240 nodes (i.e. countries) in the global food trade network, 123 nodes (i.e. CFS areas) in the national food flow network, and 163 nodes (i.e. households) in the village food flow network. Note that the ‘village’ provided in Table 1 is Kaktovic for comparison purposes. Kaktovic is representative of the other two Alaskan villages as shown in Table 2. Note that the number of nodes is constant across food and non-food flow networks. This indicates that all nodes trade both food and non-food. However, the number of links varies between commodity classes. Global and national domains have more non-food links, while the village domain has more food links.

**Table 1:** Summary statistics for commodity flow networks. Statistics are presented for undirected food and non-food networks across spatial scales.

	Global	National	Village
Food			
# Nodes	240	123	163
# Links	13,438	3,002	628
Density	0.47	0.40	0.05
Mass [kg]	1.67 x 10^12^	0.41 x 10^9^	68,117
Value [$]	1.07 x 10^12^	0.38 x 10^12^	
Non-food			
# Nodes	240	123	163
# Links	17,160	5,824	384
Density	0.60	0.66	0.03
Mass [kg]	9.28 x 10^12^	2.62 x 10^9^	66,789
Value [$]	12.30 x 10^12^	3.41 x 10^12^	

**Table 2:** Summary statistics for village commodity flow systems. Statistics are presented for undirected food and non-food networks.

	Venetie	Wainwright	Kaktovi
Food			
# Nodes	205	217	163
# Links	560	1,063	628
Density	0.03	0.05	0.05
Mass [kg]	21,947	118,498	68,117
Non-food			
# Nodes	205	217	163
# Links	277	570	384
Density	0.01	0.02	0.03
Mass [kg]	13,862	190,425	66,789

Network density decreases with spatial scale for food commodities. This means that the fraction of realized to potential links declines as the spatial scale decreases. This relationship hints at scale dependence in food flow networks. Yet, density does not follow this clear pattern for non-food commodities, in which the density of the national scale is actually greater than it is at the global scale. Density of the village scale is dramatically lower than it is for the global and national scale. This is true for both food and non-food flows.

The mass and value of global trade is on the same order of magnitude for both food and non-food. This is quite surprising given the relatively low value of food commodities. Interestingly, national commodity flows in the United States are higher in value than they are mass for both food and non-food commodities. In fact, value flows within the United States are comparable to the entirety of the global trade system. This indicates that roughly the same value of commodities flows within a country as across all national borders, highlighting the importance of considering sub-national commodity fluxes.

### 3.2 Network distributions and parameters

#### 3.2.1 Network connectivity

**Fig 2:**
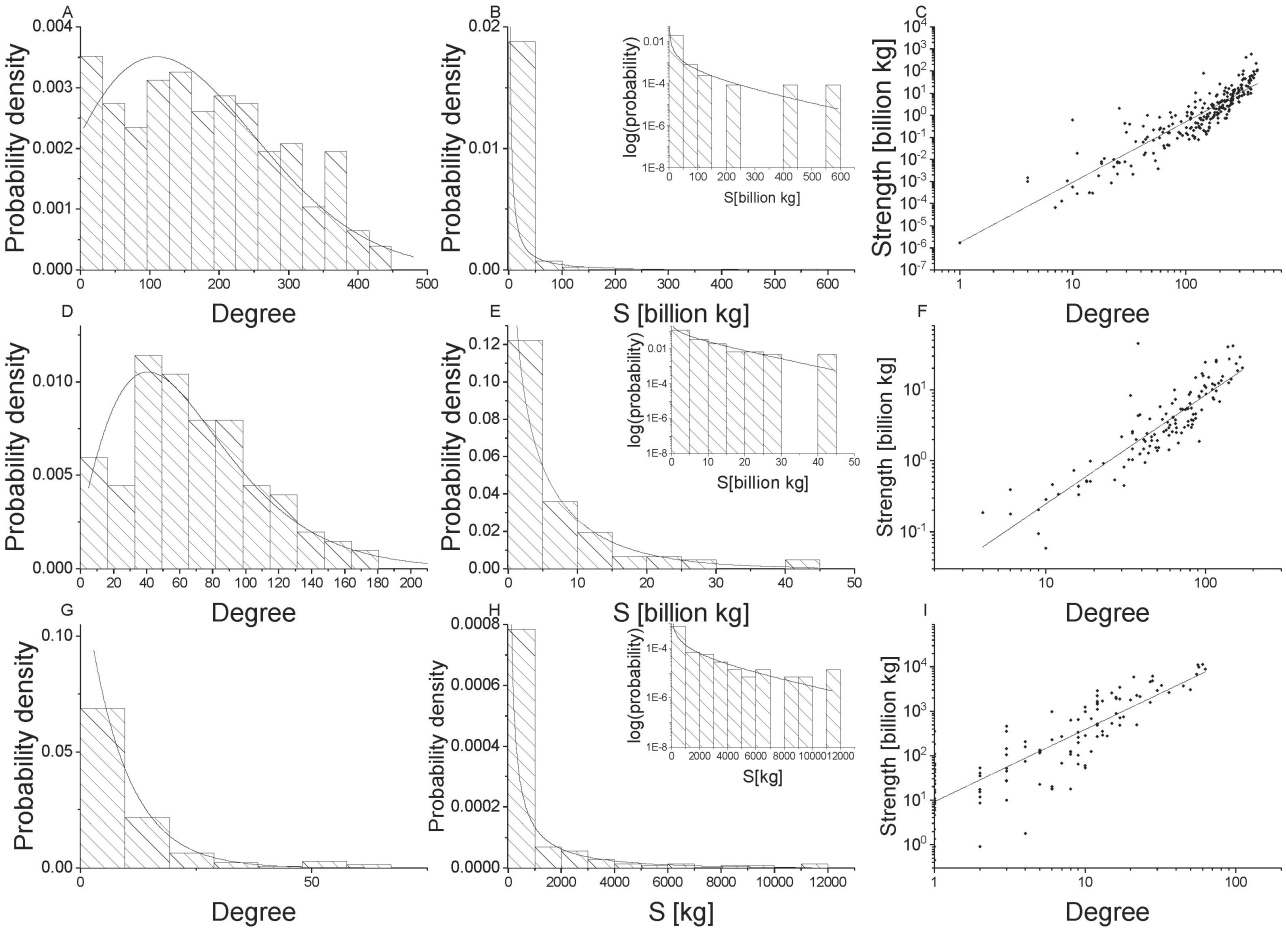
Network properties for undirected food flow networks. Global scale is shown in the top row (Panels A, B, C), national scale is shown in the middle row (Panels D, E, F), and village scale is shown in the bottom row (Panels G, H, I). Node degree distributions with generalized exponential distributions fit to the data are shown in the first column (Panel A, D, G), node strength [kg] distributions with gamma distributions fit to the data are shown in the second column (Panels B, E, H), and power law relationships for node strength versus degree are shown in the third column (Panels C, F, I).

Fig 2A, 2D and 2G present the degree distributions of undirected food flows across spatial scales. We fit a generalized exponential distribution to the node degree (*k*) distribution at each scale. The generalized exponential probability density function is given by:

$$\begin{array}{c} f_{ij}\left(x,a,b,c,d,e\right)=\frac{f(\frac{x-d}{e},a,b,c)}{d} \end{array}$$ (1)

where *a*, *b*, and *c* are shape parameters, *d* is a location parameter, and *e* is a scale parameter. The function can also be expressed in the ‘standardized’ form:

$$\begin{array}{c} f_{ij}\left(x,a,b,c\right)=\left[a+b\left(1-e^{-cx}\right)\right]exp\left[-ax-bx+\frac{b}{c}\left(1-e^{-cx}\right)\right] \end{array}$$ (2)

Here, *f*(*x*, *a*, *b*, *c*, *location*, *scale*) is equivalent to *f*(*x*, *a*, *b*, *c*)/*scale* with $y=\frac{\left(x-location\right)}{scale}$, where *y* is the imaginary value of discrete data being continuous [37, 38]. So, the generalized exponential distribution used here has 5 parameters.

**Fig 3:**
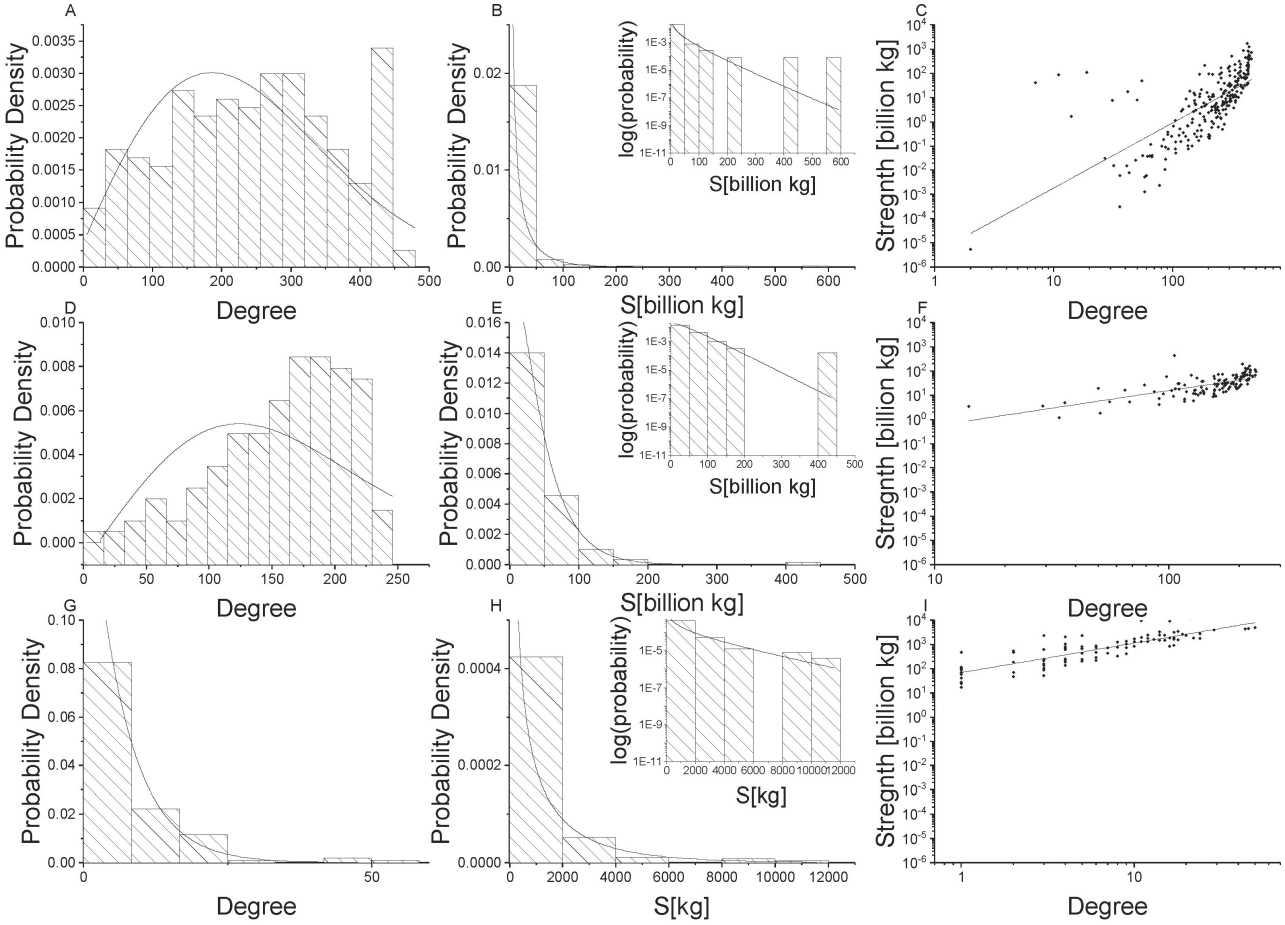
Network properties for undirected non-food flow networks. Global scale is shown in the top row (Panels A, B, C), national scale is shown in the middle row (Panels D, E, F), and village scale is shown in the bottom row (Panels G, H, I). Node degree distributions with generalized exponential distributions fit to the data are shown in the first column (Panel A, D, G), node strength [kg] distributions with gamma distributions fit to the data are shown in the second column (Panels B, E, H), and power law relationships for node strength versus degree are shown in the third column (Panels C, F, I).

Fig 2A, 2D and 2G illustrate that undirected food flow node degree distributions are well fit by a generalized exponential distribution across all spatial scales. However, Fig 3A, 3D and 3G indicate that non-food flows are not well fit by the generalized exponential distribution. In particular, the right tail of the histogram for global and national connectivity exhibit higher values than can be captured by the generalized exponential distribution. This indicates that food and non-food commodity flows exhibit different network structures, likely due to differences in the underlying driving mechanisms. The generalized exponential distribution parameters for undirected food and non-food networks are provided by spatial scale in Table 3.

**Table 3:** Parameters for undirected food and non-food commodity flow networks. Note that a generalized exponential distribution is fit to node degree, Gamma distribution is fit to node strength, and a power law is fit to node degree versus strength.

	Global	National	Village
UNDIRECTED FOOD			
Generalized Exponential Distribution			
*a*	8.45*e* − 003	8.31*e* − 003	2.78*e* + 00
*b*	7.80*e* − 001	1.61*e* + 00	2.25*e* − 011
*c*	5.68*e* − 004	2.35*e* − 003	5.30*e* − 001
*location*	0	0	0
*scale*	3.94*e* + 00	3.69*e* + 00	2.66*e* + 001
*KL* − *divergence*	0.54	0.39	1.22
Gamma Distribution			
*α*	2.62*e* − 01	8.28*e* − 01	2.13*e* − 01
*θ*	5.33*e* + 01	8.15*e* + 00	4.44*e* + 03
*KL* − *divergence*	1.17e-01	4.37e-02	1.72e-01
Power Law Fit			
*a*	− 5.77 ± 0.18	− 2.14 ± 0.13	0.96 ± 0.07
*b*	2.73 ± 0.09	1.53 ± 0.07	1.63 ± 0.09
R^2^	0.81	0.78	0.72
NON-FOOD			
Generalized Exponential Distribution			
*a*	3.61*e* − 04	2.89*e* − 06	2.25*e* + 00
*b*	9.39*e* − 02	4.28*e* − 01	1.01*e* − 11
*c*	5.88*e* − 03	3.16*e* − 03	4.49*e* − 01
*location*	0	0	0
*scale*	4.30*e* + 00	4.33*e* + 00	1.64*e* + 01
*KL* − *divergence*	0.33	0.23	1.16
Gamma Distribution			
*α*	2.70*e* − 01	1.30*e* + 00	3.60*e* − 01
*θ*	2.86*e* + 02	3.26*e* + 01	2.97*e* + 03
*KL* − *divergence*	1.17e-01	9.24e-03	6.13e-02
Power Law Fit			
*a*	− 5.43 ± 0.43	− 1.72 ± 0.30	1.84 ± 0.05
*b*	2.69 ± 0.18	1.45 ± 0.14	1.22 ± 0.07
R^2^	0.47	0.48	0.73

**Fig 4:**
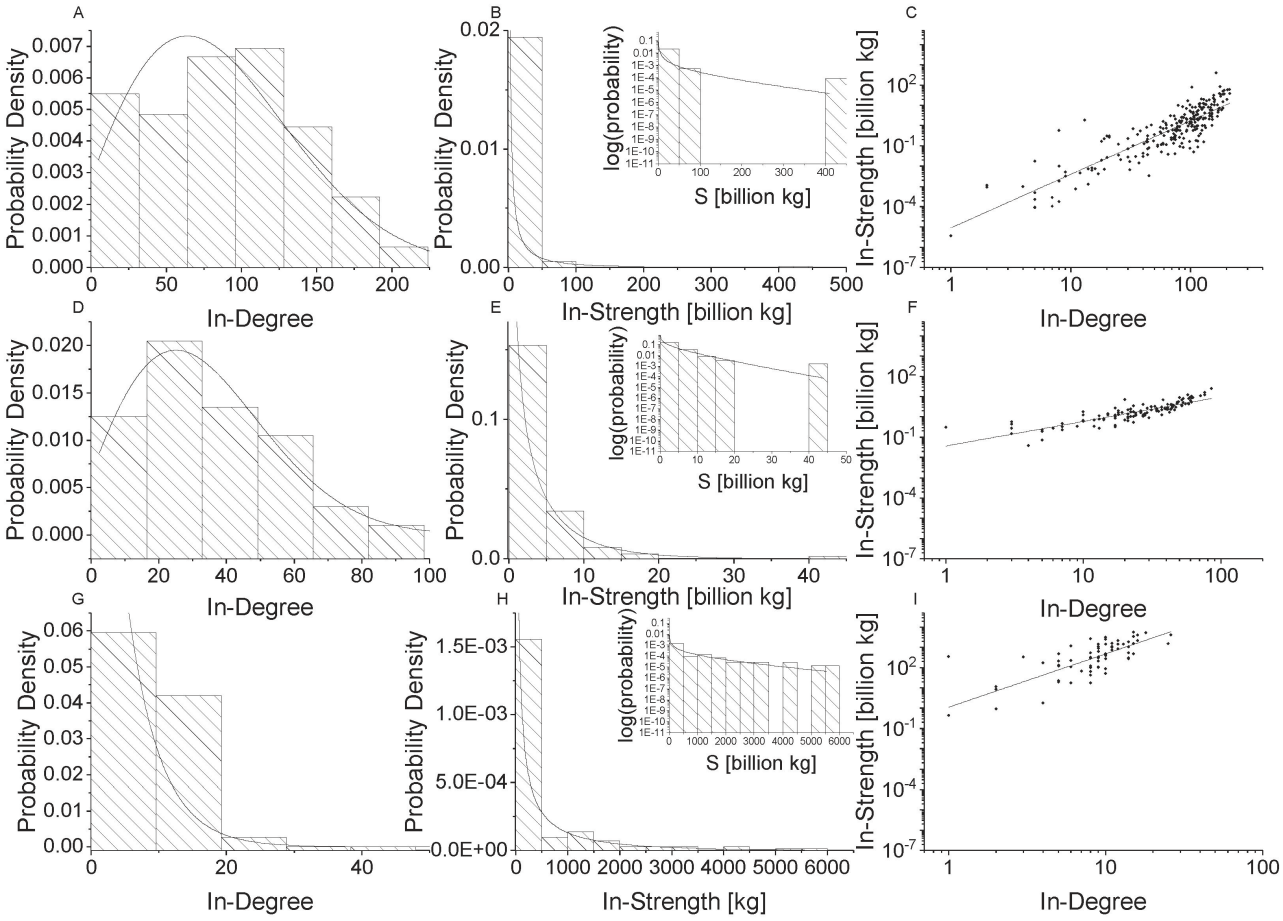
Network properties for in-food flow networks. Global scale is shown in the top row (Panels A, B, C), national scale is shown in the middle row (Panels D, E, F), and village scale is shown in the bottom row (Panels G, H, I). Node in-degree distributions with generalized exponential distributions fit to the data are shown in the first column (Panel A, D, G), node in-strength [kg] distributions with gamma distributions fit to the data are shown in the second column (Panels B, E, H), and power law relationships for node in-strength versus in-degree are shown in the third column (Panels C, F, I).

**Fig 5:**
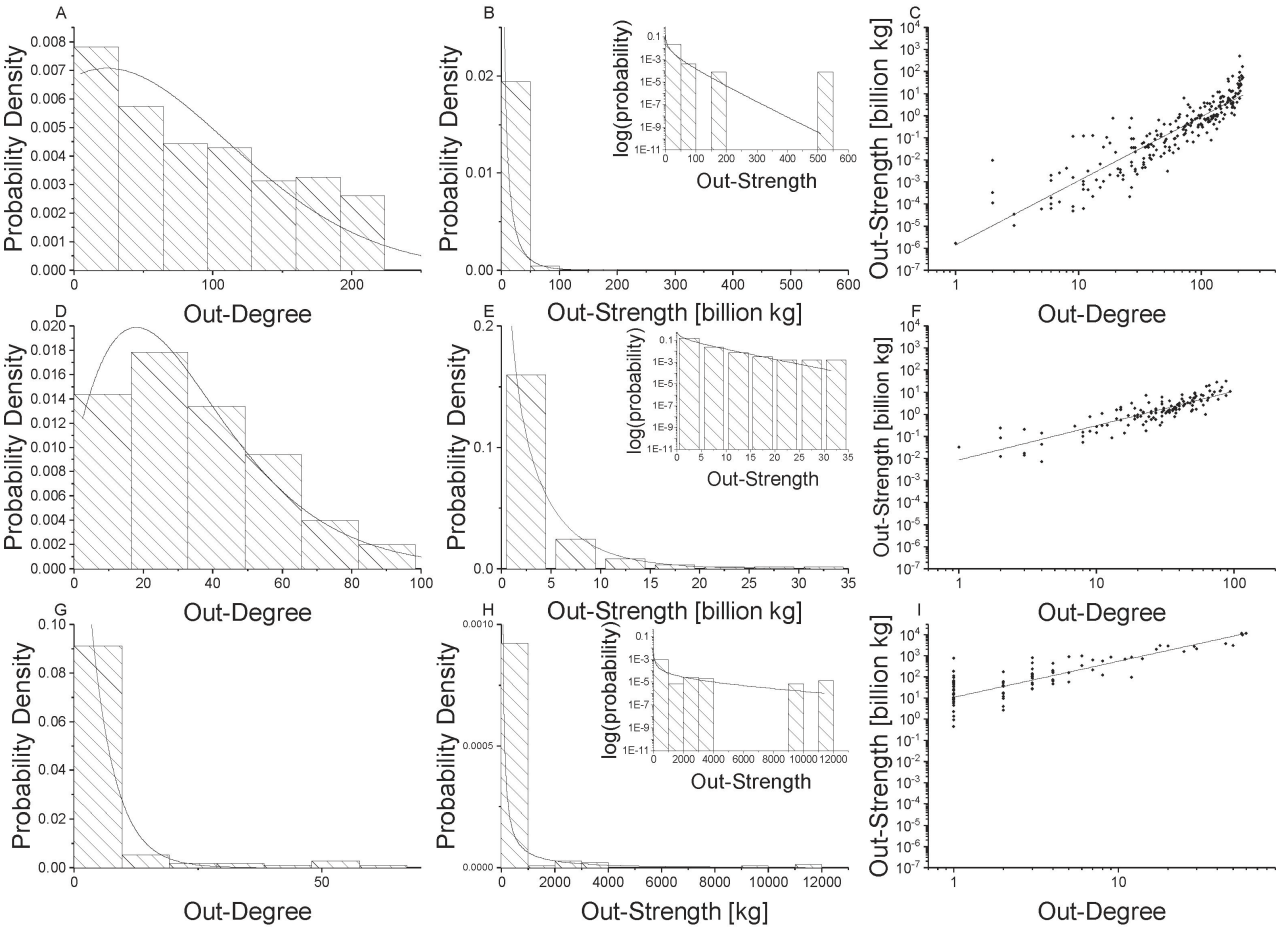
Network properties for out-food flow networks. Global scale is shown in the top row (Panels A, B, C), national scale is shown in the middle row (Panels D, E, F), and village scale is shown in the bottom row (Panels G, H, I). Node out-degree distributions with generalized exponential distributions fit to the data are shown in the first column (Panel A, D, G), node out-strength [kg] distributions with gamma distributions fit to the data are shown in the second column (Panels B, E, H), and power law relationships for node out-strength versus out-degree are shown in the third column (Panels C, F, I).

The generalized exponential distribution also provides a good fit to the connectivity of food flow networks with direction and value weights. When direction is taken into account, food flows are still well fit by the generalized exponential distribution. To see this, refer to Figs 4A, 4D, 4G, 5A, 5D and 5G. This indicates that food flow network connectivity can be modeled with the same distribution with or without consideration of flow directionality. This is despite the fact that the tails of in- and out-degree distributions are quite different. The distributions of out-degree are more right skewed compared to the in-degree distributions across all scales. This pattern has also been pointed out by Konar et al (2011) [27]. This indicates that countries have many export trade partners, while they tend to import from just a few trade partners. This makes sense under the principle of economic specialization, in which a location that is efficient at producing a given commodity will specialize in it’s production and export. Conversely, if a nation imports a given commodity, it will likely be able to meet its demand for this commodity in a few trade relationships.

**Fig 6:**
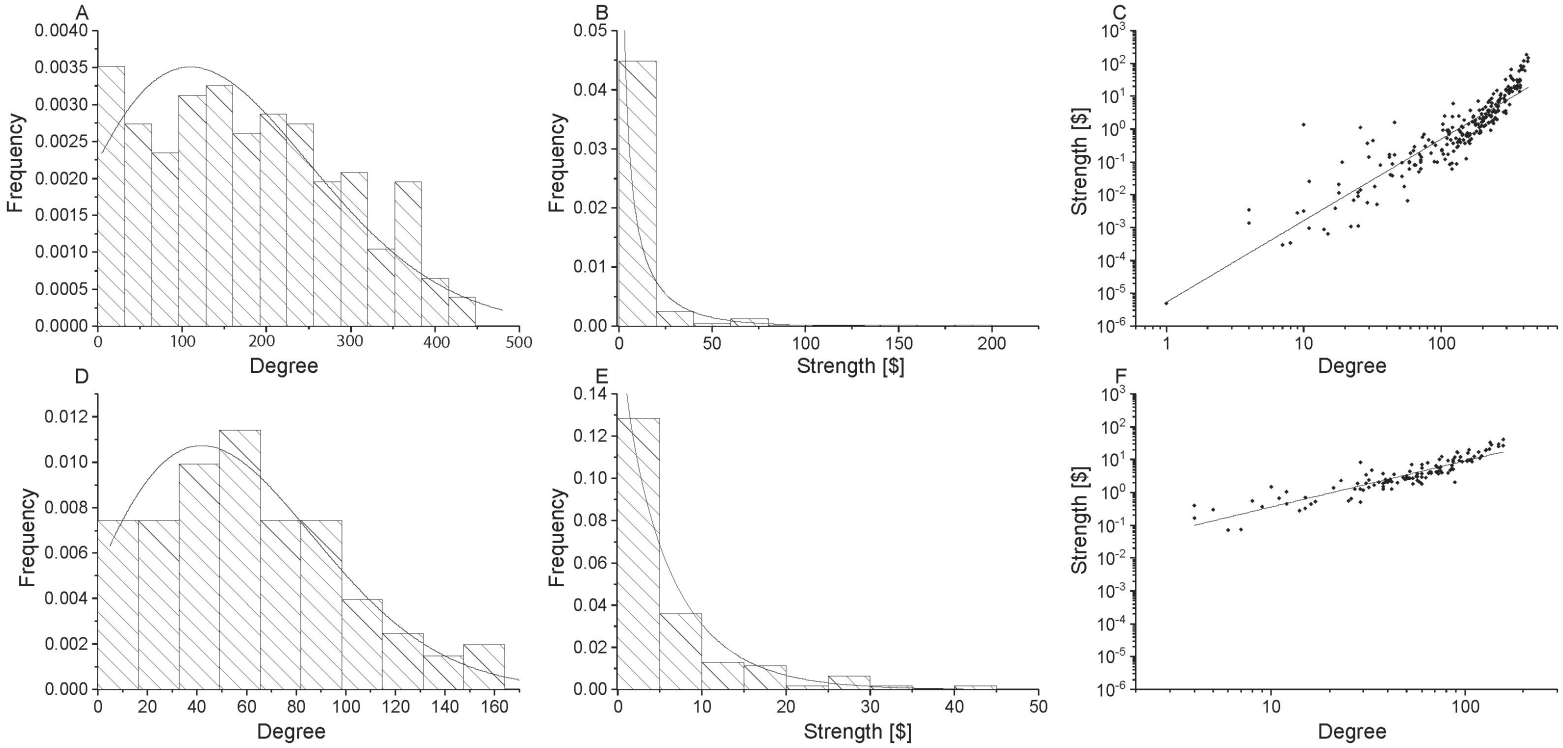
Network properties for global (top row) and national (bottom row) commodity flow networks by value [$]. Node degree distributions with generalized exponential binomial distributions fit to the data are shown in the first column (Panel A, D), node strength distributions with gamma distributions fit to the data are shown in the second column (Panels B, E), and power law relationships for node strength versus degree are shown in the third column (Panels C, F).

**Fig 7:**
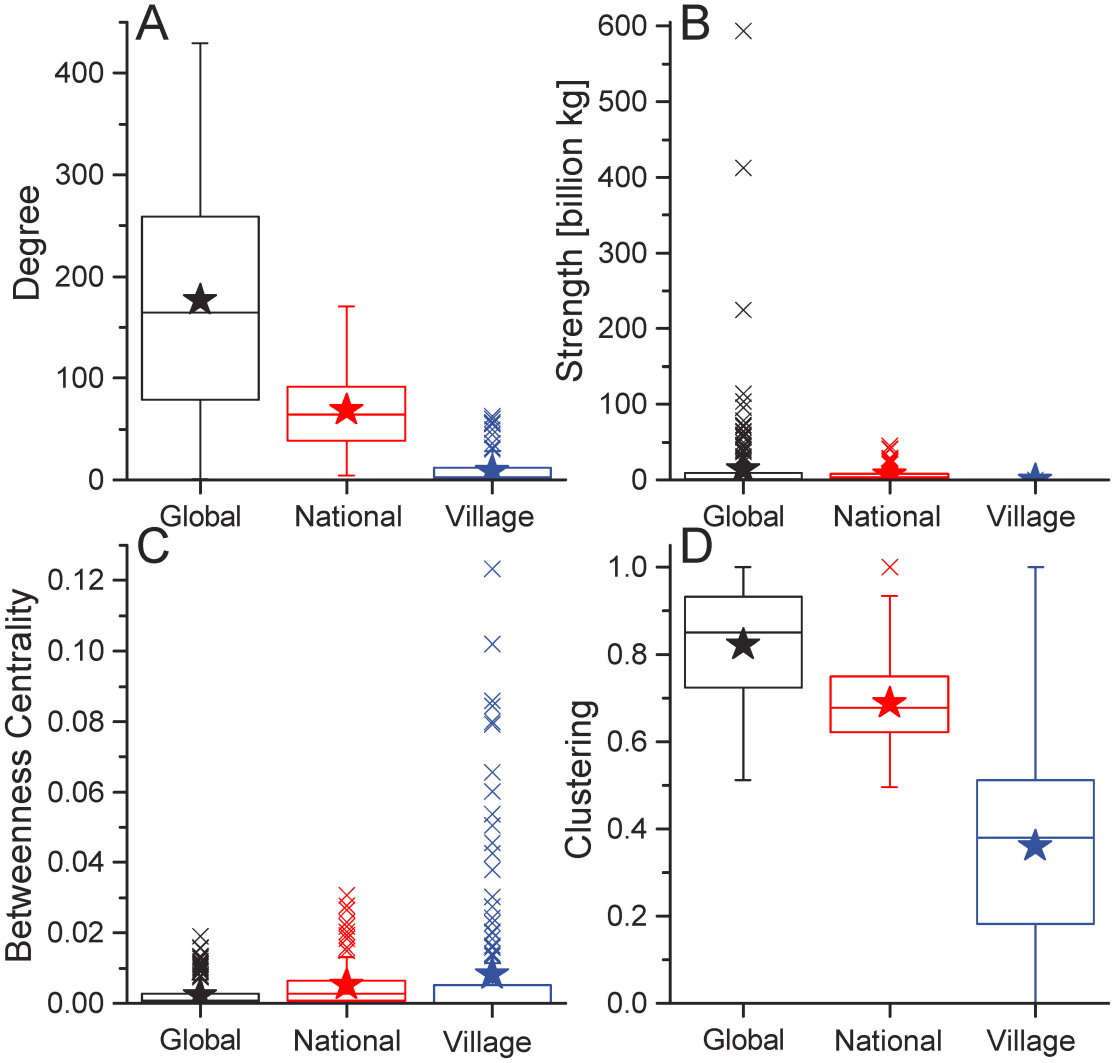
Network properties for undirected food flow networks of all villages. Kaktovic is shown in the top row (Panels A, B, C), Venetie is shown in the middle row (Panels D, E, F), and Wainwright is shown in the bottom row (Panels G, H, I). Node degree distributions with generalized exponential distributions fit to the data are shown in the first column (Panel A, D, G), node strength distributions with gamma distributions fit to the data are shown in the second column (Panels B, E, H), and power law relationships for node strength versus degree are shown in the third column (Panels C, F, I).

**Fig 8:**
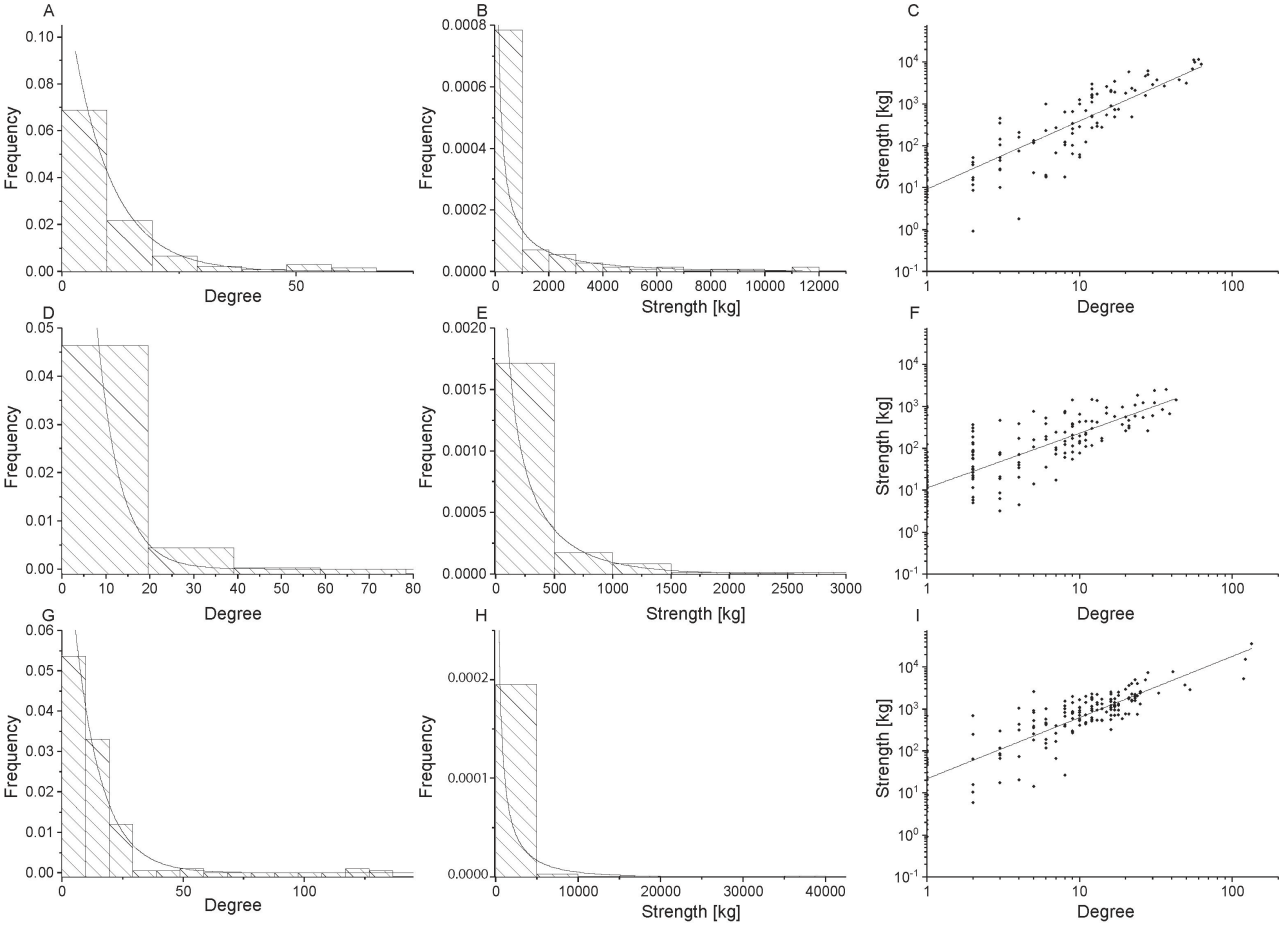
Network distributions for undirected food flows by spatial scale. Node degree (Panel A), strength (Panel B), betweenness centrality (Panel C), and clustering (Panel D) are shown for global, national, and village spatial scales. Box-whisker plots present the median (box line), interquartile range (box), mean (star), and outliers (“x”).

Fig 6A, 6D and 6G illustrate that the generalized exponential distribution fits the connectivity structure of food flows well when value [$] weights are assigned rather than mass fluxes [kg]. This is what we would expect, since link weights are not considered in the connectivity structure, but it is good to empirically determine this. Additionally, all three Alaskan villages exhibit the same network properties (refer to Fig 7). However, Fig 8A illustrates that mean node connectivity decreases with spatial scale, despite the fact that the statistical distribution remains the same.

The generalized exponential distribution is an extension of the exponential distribution, much like the Gamma or Weibull distributions [39]. A more parsimonious distribution (i.e. such as the Gamma or Weibull distributions) would be desirable to fit to our data. However, rather than model parsimony, we selected the model with the best fit to the data. In the end, the generalized exponential distribution has qualities that are beneficial for our setting. The generalized exponential distribution is an extension of the bivariate exponential distribution (BVE). So, in order to understand the generalized exponential distribution, we first need to understand the BVE model. The BVE model has been used to ascertain the failure time of two plants that use common components. For example, both plants use engines produced by the same company. This means that any failure related to the common component will be correlated across plants. At the same, these two plants also use different components. Thus, this introduces potential independent reasons for failure. In the BVE model, the waiting time for failure does not change with the passing of time (due to Poisson process assumption). This gives a memoryless property to the BVE model.

The generalized exponential distribution makes sense as a representation of trade systems. Replace one plant in the model with one trading node, and the other plant with all other nodes in the trading system. Then, assume that these two entities will keep producing one new link per time unit. A failure occurs when a link in the trade system is broken. In our commodity flow systems, failure could arise either from a common component (for example, correlated disaster in the production of a commodity in both nodes due to weather) or from different components. The generalized exponential model is an adaption of the BVE model such that failure becomes more likely as time goes by. In other words, the generalized exponential distribution is not memoryless, such that shocks accumulate in time [37]. This makes sense, as disruptions to trade would likely be taken into account by trading partners going forward. This feature of the generalized exponential distribution leads to an increased failure rate when exposed to external shocks. Additionally, the generalized exponential distribution enables the shock arrival rates to be separately identified from their impacts [37]. For this reason, the generalized exponential distribution—which provides the best fit to our data—also possesses properties that likely capture important aspects of trade systems. This feature will likely prove useful in future research that aims to model the impact of shocks to the global food trade system.

#### 3.2.2 Network mass fluxes

The distributions of mass transfers for undirected food flow networks are provided in Fig 2B, 2E and 2H. We fit a Gamma distribution to the node strength (*s*) distribution at each scale. The Gamma probability distribution function is:

$$\begin{array}{c} \frac{1}{\Gamma \left(\alpha \right)\theta^{\alpha }}x^{\alpha -1}e^{-\frac{x}{\theta }} \end{array}$$ (3)

where Γ is the Gamma function, *α* is the shape parameter, and *θ* is the scale parameter of the Gamma distribution. Fig 2B, 2E and 2H show that node mass flux, or strength (*s*), distributions for undirected food flow networks are fit well by a Gamma distribution across all spatial scales. This highlights that these networks are much more heterogeneous in terms of their mass fluxes than connectivity structure.

The Gamma(shape, success rate) distribution is generated from a poisson process. Conceptually, the gamma distribution can be explained as commodity shipments to meet a “shape” amount of need. The chance that a unit of transported commodity will be successful and meet one unit of need is the “success” rate. The Gamma distribution parameters (*α*, *θ*) are provided for undirected food and non-food commodities in Table 3. Graphs of the Gamma distribution fit to non-food flow networks are provided in Fig 3. It is clear that the Gamma distribution does not fit non-food as well as it does food commodities. The difference in Gamma distribution fits between food and non-food is particularly clear when comparing the log-lin insets in Fig 2B, 2E and 2H with Fig 3B, 3E and 3H.

Figs 4 and 5 show the Gamma distribution fit to directed food flow networks. Fig 4 shows that the Gamma distribution fits food-in mass fluxes well. The Gamma distribution also fits the food-out mass fluxes well across scales. However, the Gamma distribution underestimates the largest food exporter at the global scale. To see this, refer to the inset in Fig 5B. However, note that the statistical fit still performs well (i.e. see the KL-divergence value in Table 4).

**Table 4:** Parameters for in-food and out-food commodity flow networks. Note that a generalized exponential distribution is fit to node degree, Gamma distribution is fit to node strength, and a power law is fit to node degree versus strength.

	Global	National	Village
IN-FOOD			
Generalized Exponential Distribution			
*a*	5.39*E* − 03	1.11*E* − 02	1.72*E* + 00
*b*	2.18*E* + 00	4.71*E* + 00	3.95*E* − 10
*c*	1.54*E* − 04	5.88*E* − 04	6.99*E* − 01
*location*	0	0	0
*scale*	1.58*E* + 00	1.67*E* + 00	8.23*E* + 00
*KL* − *divergence*	8.75*E* − 02	8.10E-02	4.65E-01
Gamma Distribution			
*α*	5.88*E* − 02	4.51*E* − 01	2.26*E* − 01
*θ*	1.19*E* + 02	7.47*E* + 00	2.09*E* + 03
*KL* − *divergence*	2.30E-01	2.80E-02	1.80E-01
Power Law Fit			
*a*	− 5.05 ± 0.18	− 1.44 ± 0.09	0.05 ± 0.18
*b*	2.65 ± 0.10	1.22 ± 0.07	2.63 ± 0.20
R^2^	0.75	0.74	0.70
OUT-FOOD			
Generalized Exponential Distribution			
*a*	4.92*E* − 12	4.03*E* − 02	2.32*E* + 00
*b*	3.14*E* − 02	7.09*E* − 01	4.52*E* − 11
*c*	1.27*E* + 00	2.00*E* − 02	1.98*E* + 00
*location*	0	0	0
*scale*	2.71*E* + 00	4.02*E* + 00	1.11*E* + 01
*KL* − *divergence*	0.81	0.52	1.69
Gamma Distribution			
*α*	2.03*E* − 01	6.42*E* − 01	8.31*E* − 02
*θ*	3.44*E* + 01	5.25*E* + 00	5.70*E* + 03
*KL* − *divergence*	6.70E-02	3.14E-02	5.96E-01
Power Law Fit			
*a*	− 5.85 ± 0.17	− 2.07 ± 0.12	1.03 ± 0.07
*b*	2.90 ± 0.09	1.56 ± 0.08	1.70 ± 0.11
R^2^	0.81	0.75	0.66

The outlier in Fig 5B is the country that exports the most food in mass, which is China. In 2009, China exported 5.11 x 10^11^ kg of food. The country that exports the second largest quantity of food is the U.S., with a total food export of 1.74 x 10^11^ kg. It is possible that 2009 is an anomalous year (for example, due to the Great Recession in the U.S.). Note that, here, ‘food’ encapsulates more than raw agricultural commodities, which the U.S. has been shown to export more than China (e.g. shown in Dalin et al., 2012). The value of the food export of the U.S. is larger than that of China (i.e. U.S. food export value is $1.02 x 10^11^; China food export value is $3.91 x 10^10^), although the value of non-food export is larger in China (i.e. China non-food export value is $1.35 x 10^12^; U.S. non-food export value is $9.18 x 10^11^). China and U.S. food export values are broken down by HS code in Table 5.

**Table 5:** Total export quantities for China and the United States in 2009. Quantities are shown by HS code for the food commodities (i.e. HS codes 1-24). HS codes 25-97 are non-food commodities. Note that the U.S. exports an order of magnitude more cereals and oil seed crops than does China, but that China exports an order of magnitude more in other food categories, such as beverages, spirits, and vinegar (with a high mass). Total values are shown for both food and non-food exports.

HS code	Item	China export [kg]	China export [$]	U.S. export [kg]	U.S. export [$]
1	Animals, live	1.99E+08	4.70E+08	6.32E+07	8.16E+08
2	Meat & edible meat offal	7.06E+08	1.18E+09	6.34E+09	1.10E+10
3	Fish & crustaceans	2.25E+09	7.04E+09	1.25E+09	3.96E+09
4	Dairy produce	1.98E+08	3.66E+08	1.13E+09	2.04E+09
5	Animal originated products	2.02E+08	1.36E+09	3.29E+08	7.48E+08
6	Trees & other plants	8.90E+07	2.47E+08	9.90E+07	3.75E+08
7	Vegetables	6.38E+09	4.69E+09	3.43E+09	3.30E+09
8	Fruit & nuts	3.75E+09	2.90E+09	4.64E+09	8.44E+09
9	Coffee & tea	8.78E+08	1.40E+09	1.33E+08	7.09E+08
10	Cereals	1.21E+09	6.82E+08	7.58E+10	1.87E+10
11	Products of the milling industry	1.03E+09	5.36E+08	1.79E+09	1.01E+09
12	Oil seeds	1.54E+09	2.06E+09	4.48E+10	1.96E+10
13	Lac, gums, resins	4.68E+07	4.84E+08	4.82E+07	5.36E+08
14	Vegetable products	1.29E+08	9.87E+07	8.88E+07	6.85E+07
15	Animal or vegetable oils	2.90E+08	4.16E+08	3.97E+09	3.66E+09
16	Animal preparations	9.47E+08	4.14E+09	4.92E+08	1.68E+09
17	Sugars	1.06E+09	8.66E+08	2.05E+09	1.24E+09
18	Cocoa	4.50E+07	1.37E+08	2.89E+08	1.12E+09
19	Preparations of cereals	7.45E+08	1.08E+09	1.47E+09	2.93E+09
20	Preparations of vegetables	4.19E+09	4.70E+09	2.59E+09	3.60E+09
21	Edible preparations	7.01E+08	1.36E+09	1.43E+09	5.28E+09
22	Beverages, spirits & vinegar	4.81E+11	1.00E+09	2.40E+09	3.85E+09
23	Residues	3.64E+09	1.84E+09	1.89E+10	7.49E+09
24	Tobacco	2.67E+08	1.08E+09	1.87E+08	1.98E+09
	Total food	5.11E+11	4.02E+10	1.74E+11	1.04E+11
	Total non-food	3.54E+11	1.35E+12	4.14E+11	9.18E+11

Node strength distributions in value [$] weights are provided in Fig 6 for the global and national spatial scales. These figures all indicate that the Gamma distribution provides a good fit to the intensity of commodity flows across commodity types, with or without directionality, and for both mass and value weighting schemes. Fig 8B shows that nodal mass flux decrease with spatial scale, as we would expect, even though a Gamma distribution provides a suitable model across scales.

Here, we have shown that the Gamma distribution captures nodal strength distributions across all flow networks reasonably well. However, the Gamma distribution underestimates outliers in some instances; for example, missing the export dominance of China in 2009. Yet, the Gamma distribution is a flexible statistical model that is broadly representative of commodity mass fluxes. There are known properties of the Gamma distribution, which means that future efforts to model commodity fluxes may be able to benefit from these attributes. For example, the Gamma distribution has known reliability, lifetime, and hazard functions [40]. These statistical properties can be taken into account to model commodity fluxes in future research.

#### 3.2.3 Relationship between connectivity and flows

We examine the relationship between node degree and strength for undirected food flows in Fig 2C, 2F and 2I. In Fig 2C, 2F and 2I *s* is plotted against *k* for all spatial scales. The straight line relationship in log-log scale indicates that there is scale invariance between mass flows and network connectivity. Specifically, a power law relationship between nodal mass flux and connectivity is evident across all spatial scales. Thus, there is a power law relationship between *s* and *k* food flows and it is consistent across village, national, and global food networks.

A linear relationship is fit to log(*s*) and log(*k*) such that:

$$\begin{array}{c} \text{log}(s)=a+b\;\text{log}(k) \end{array}$$ (4)

The parameters of the power law relationship for undirected food and non-food flows are provided in Table 3. The statistical distribution parameters change with the scale of analysis, indicating that there is scale dependence, despite the fact that the power law exists across scales. The power law exponent is the highest for global trade (slope = 2.7; see Table 3), but is similar for national and village scales (slope = 1.5 and 1.6, respectively). The power law relationship is less clear for non-food flows. Note that the points exhibit more scatter in Fig 3 than in Fig 2. Likewise, the exponents are consistently smaller for non-food flows than for food flows. Again, this indicates that food and non-food flow networks exhibit different properties which may be due to their underlying unique attributes.

What are the implications of a power law relationship for node strength versus degree? Particularly for global trade, the high *b* value indicates that there is a strong relationship between the mass that each nation trades and its number of trade partners. In other words, the node strength grows faster than node degree, so the more trade connections a country has, the much more it is able to participate in the exchange of commodity mass. This relationship occurs in a highly nonlinear way. In this way, shocks to trade relationships may prove highly disruptive to national access to the mass of food commodities, unless trade patterns are allowed to adapt. This is another statistical attribute that future efforts to model food flows may endeavor to incorporate.

#### 3.2.4 Network clustering and centrality

The clustering coefficient enables us to evaluate the tendency of nodes in the network to form tightly connected groups. In Fig 8D, it is clear that node clustering decreases with spatial scale. In other words, nations are more likely to form ‘cliques’ than are households in a village [34]. However, node clustering decreases in a much less consistent manner than it does for degree and strength. This can be seen by the fact that the whiskers in the box-whisker plot overlap for clustering. Scale dependency in network parameters indicated by Fig 8D likely arises as a result of the aggregation process in food fluxes from smaller to larger scales of analysis.

**Fig 9:**
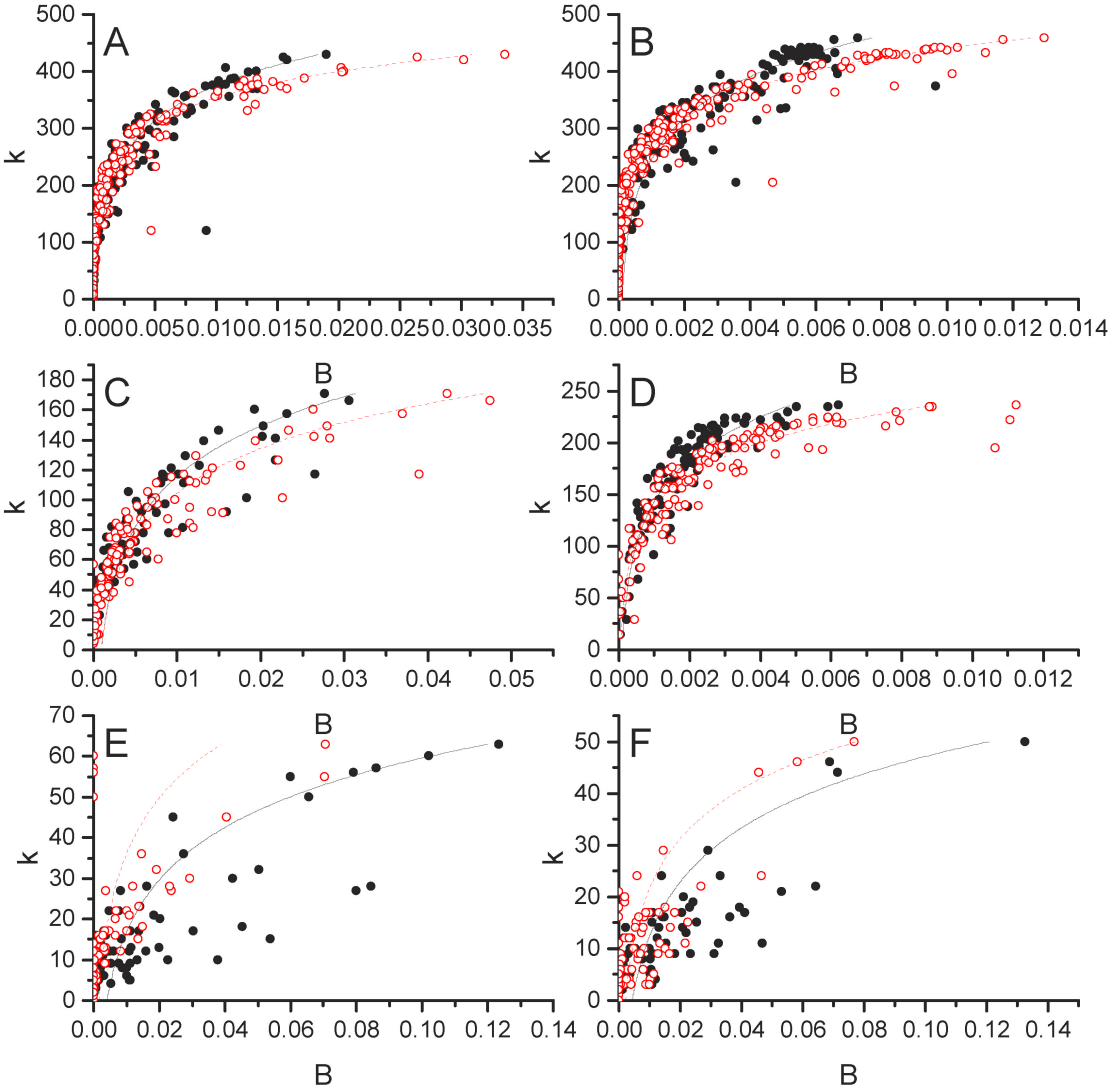
Node degree (*k*) versus betweenness centrality (*B*) for food and non-food commodity flow systems. The global scale is shown in the top row (Panels A, B), national scale is shown in the middle row (Panels C, B), and village scale is shown in the bottom row (Panels E, F). Food commodities are shown in the first column (Panels A, C, E) and non-food commodities are shown in the second column (Panels B, D, F). Red points show directed *B* and black points show undirected *B*. Note axes scales differ across panels.

Fig 9 presents the relationship between betweenness centrality (*B*) and degree (*k*) for food fluxes by spatial scale. Here, an exponential distribution is used to fit the relationship between *B* and *k*. Specifically, we fit the following function: *k* = *a* * *exp*(*b* * *B*). The shape of the *B* versus *k* relationship is consistent across scales and between food and non-food commodity groups (shown in Fig 9). The shape of the relationship is relatively similar with or without the incorporation of directionality. However, directed networks exhibit higher centrality (i.e. note the red points have larger x-axis values). In other words, certain nodes play a more central role in networks in which direction is taken into account. Interestingly, this pattern is reversed for village networks. In Fig 9E and 9F, the red and black lines flip and the black points have larger x-axis values. This means that direction is less important to node centrality at the village scale than it is at the national and global scales.

**Fig 10:**
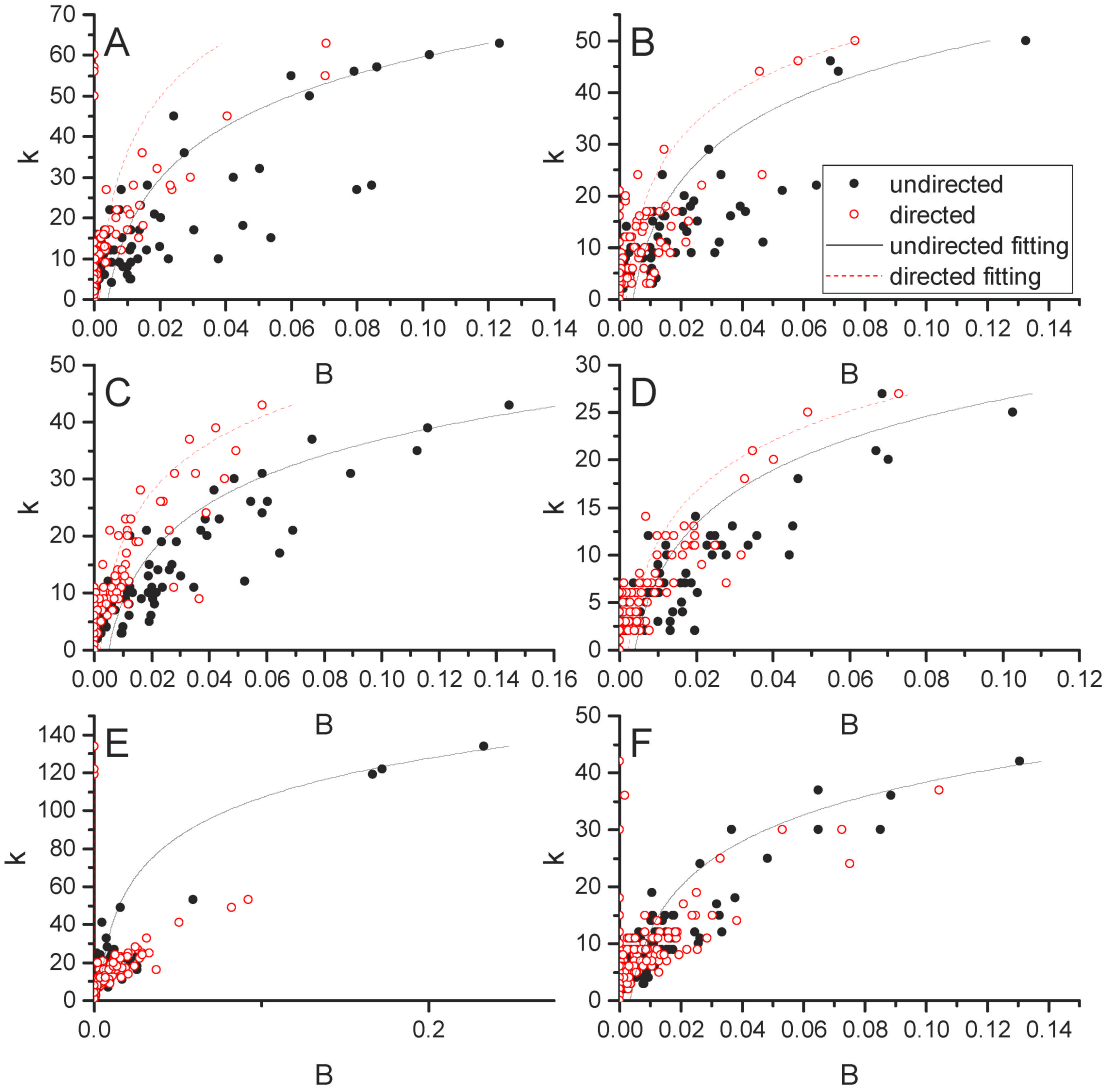
Node degree (*k*) versus betweenness centrality (*B*) for food and non-food village commodity flow systems. Kaktovic is shown in the top row (Panels A, B, C), Venetie is shown in the middle row (Panels D, E, F), and Wainwright is shown in the bottom row (Panels G, H, I). Food commodities are shown in the first column (Panels A, C, E) and non-food commodities are shown in the second column (Panels B, D, F).

The nodes that have both high *k* and *B* values are referred to as the network ‘core’. A core has been shown for global [3] and national [17] food flows. We confirm that this relationship exists for both undirected and directed food and non-food commodities (refer to Fig 9), although a core group of nodes is more pronounced for food networks. We also show that a core group of nodes exists at the smallest spatial scale. In fact, core households at the village scale exhibit the highest centrality of all food flows networks. Note that the scale on the x-axis is an order of magnitude larger for the village scale than for the global scale. This indicates that some households are more instrumental to the structure and functioning of village scale food exchanges than are countries at the global scale. The network core exists for all Alaskan villages, as shown in Fig 10.

The relationship between connectivity and centrality is similar across spatial scales. Yet, scale dependence also exists since the statistical attributes of *B* vary with spatial scale. This result is shown in Fig 8C, in which *B* increases with decreasing spatial scale. At the village scale, *B* is more concentrated amongst some households that are dramatic outliers. These *B* outliers are not evident in the global and national scales. So, some households in the village scale are more central to its food flow network than any CFS areas are to the national network or countries are to the global network. In this way, the village is more vulnerable to the removal of its core households.

## 4 Conclusion

Food flow networks may prove to be an important adaptation measure to cope with future climate and economic shocks. For example, if extreme climate events increase in frequency as projected under a changing climate, the ability to transfer commodities in both space and time may help those production locations that experience shocks to maintain consumption by importing from external sources. As such, it is essential to understand the scaling properties of food commodity flow networks so that we can understand how to model food flows and evaluate opportunities and roadblocks to the spatial and temporal redistribution of goods. Information on the scaling properties of food flow networks will also enable prediction of flows for the many locations and settings for which food transfer data is not available.

We have examined the empirical network structure of food commodity exchanges across the full spectrum of spatial scales. Village scale donations of food between household is the smallest spatial scale at which social food fluxes can occur; global scale international food trade between countries represents the largest possible spatial domain. Empirical evidence suggests that both scale dependent and invariant properties exist for food flow networks. Network parameters such as mean node connectivity, mass flux, and centrality vary with spatial scale, likely due to the aggregation process of food fluxes. Yet, we find that the statistical distribution functions of node connectivity and mass transfers are invariant across scales. Likewise, the relationship between node connectivity and mass flux exhibits a power law relationship for each spatial domain. These relationships hold for commodity fluxes weighted in both mass [kg] and value [$] units and for both undirected and directed networks. However, non-food commodities are not well fit by the same statistical distributions across spatial scales. This highlights that there are unique attributes of food transfers that lead them to have properties that are distinct to non-food.

The network structures of food flow systems provide a signature of their vulnerability and resiliency to disturbance. Extensive research has explored the implications of certain network structures for vulnerability and resiliency. For example, networks with a power law node degree distribution have been shown to be vulnerable to targeted attack, but resilient to random attack. Future research is needed to explore the implications of the statistical network distributions of food flows presented here. Scale invariant properties indicate that similar governing mechanisms are likely influencing food flows across scales, despite the fact that these systems are typically thought to arise from starkly different generative processes. We hypothesize that universal signatures of human behavior may lead to the similarities in food network statistical distributions across scales. For example, the human tendency for risk-sharing and cooperation may be an important mechanism generating the emergent food exchange patterns. Future research can build upon this work by modeling network formation processes and estimating food flows at resolutions lacking data.
